# The Fate of Water in Hydrogen‐Based Iron Oxide Reduction

**DOI:** 10.1002/advs.202300626

**Published:** 2023-06-08

**Authors:** Ayman A. El‐Zoka, Leigh T. Stephenson, Se‐Ho Kim, Baptiste Gault, Dierk Raabe

**Affiliations:** ^1^ Max‐Planck‐Institut für Eisenforschung Max‐Planck‐Strasse 1 40237 Düsseldorf Germany; ^2^ Department of Materials Royal School of Mines Imperial College London SW7 2AZ UK; ^3^ Department of Materials Science and Engineering Korea University Seoul 02841 Republic of Korea

**Keywords:** atom probe tomography, gas–solid reactions, green steel, hydrogen metal oxide reduction

## Abstract

Gas–solid reactions are important for many redox processes that underpin the energy and sustainability transition. The specific case of hydrogen‐based iron oxide reduction is the foundation to render the global steel industry fossil‐free, an essential target as iron production is the largest single industrial emitter of carbon dioxide. This perception of gas–solid reactions has not only been limited by the availability of state‐of‐the‐art techniques which can delve into the structure and chemistry of reacted solids, but one continues to miss an important reaction partner that defines the thermodynamics and kinetics of gas phase reactions: the gas molecules. In this investigation, cryogenic‐atom probe tomography is used to study the quasi in situ evolution of iron oxide in the solid and gas phases of the direct reduction of iron oxide by deuterium gas at 700°C. So far several unknown atomic‐scale characteristics are observed, including, D_2_ accumulation at the reaction interface; formation of a core (wüstite)‐shell (iron) structure; inbound diffusion of D through the iron layer and partitioning of D among phases and defects; outbound diffusion of oxygen through the wüstite and/or through the iron to the next free available inner/outer surface; and the internal formation of heavy nano‐water droplets at nano‐pores.

## Graphical abstract

What happens to iron oxide after a mere 10 s of reduction in hydrogen? The authors are now able to examine the atomic‐scale chemical changes happening, and reduction interfaces forming by freezing oxides and counting atoms, using cryo‐atom probe tomography. One can also “see” what happens in the gas phase to the hydrogen, and the fate of the water forming during reduction.

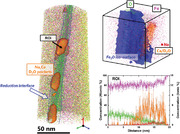

## 1 Introduction

Iron is one of the most abundant elements in the earth's crust and in many stellar objects. It has several oxidation states, mainly Fe(II) and Fe(III), and crystallizes in different oxides such as hematite, magnetite, maghemite, or wüstite. The reduction of iron oxides to iron, and the reverse process, that is, oxidation, are both redox reactions of high relevance to the catalytic synthesis of ammonia^[^ ^1^ ^]^ and hydrocarbons;^[^ ^2^ ^]^ planetary morphogenesis;^[^ ^3^ ^]^ magnets in structural biology;^[^ ^4^ ^]^ biochemical reactions, for example, as in hemoglobin;^[^ ^5^ ^]^ medical applications such as imaging,^[^ ^6^ ^]^ cell activation, cancer therapy;^[^ ^7^ ^]^ metallurgy,^[^ ^8^ ^]^ as well as waste‐ and groundwater treatment,^[^ ^9^ ^]^ to name but a few topics.

Here we focus specifically on the direct reduction of iron oxide by hydrogen, motivated by a recent surge in interest in moving away from the conventional carbon‐based production of iron from its ores, that is, among the largest single source of industrial carbon dioxide emissions responsible for climate change.^[^ ^10^, ^11^, ^12^, ^13^, ^14^ ^]^ Many studies have addressed the thermodynamic and kinetic features of the underlying reactions,^[^ ^15^, ^16^, ^17^, ^18^ ^]^ but little is known about the formation, location, and role of the final reaction product, namely, water.^[^ ^19^ ^]^

Addressing this fundamental question is essential for understanding the kinetics and achievable metallization of such redox reactions since water is expected to form at inner and outer surfaces, meaning that oxygen and hydrogen must first diffuse and then react.^[^ ^20^, ^21^ ^]^ Yet, until now, chemically and spatially resolved information on where this recombination takes place, and in which state(s) the water forms, diffuses, or gets stored remains unknown.^[^ ^22^, ^23^ ^]^ For a better understanding of these storage and transport mechanisms it is relevant to know in which way the water resides on or in the solids and what the associated diffusion mechanisms are. The many lattice defects in the partially reduced solids, such as phase boundaries, grain boundaries, dislocations, and vacancies, are likely also relevant for the diffusion and nucleation of water.^[^ ^24^, ^25^, ^26^, ^27^ ^]^

It is not only important to study how and where the water can form but also how it is removed from or stored at the reaction front. Also, the role of water in porosity formation (oxide reduction is associated with large mass loss and gain in free volume in the solid) and as an interface layer that potentially blocks further reduction is also not yet fully understood.^[^ ^15^ ^]^ It is worth noting in that context, that the presence of water can lead to undesirable re‐oxidation effects at the reaction front which can again have a high impact on the overall kinetics.^[^ ^28^ ^]^ Zhou et al.^[^ ^29^ ^]^ have recently shown in a combined experimental and theoretical study that a certain amount of the water is indeed not removed from the reaction zone via free surfaces but instead is stored inside the pores in the reduced iron. They found that many of these pores are not interconnected, so the water that becomes trapped in them cannot diffuse outbound to be released from the reaction front. A coupled mechano–chemical phase‐field simulation further revealed that a substantial water pressure of up to ≈ 4–5 atmospheres (≈0.5 MPa) can build up in these pores. This effect leads to the re‐oxidation of the surrounding metal, as proven by local diffraction experiments. This re‐oxidation effect translates to a corresponding loss in the overall metallization degree that can be achieved by a direct reduction reactor for making sustainable iron.^[^ ^30^ ^]^ According to their calculations, the affected re‐oxidized volume around the pores can amount to up to 5%. This is a large amount of unreduced material when considering the annual steel production of currently ≈ 2 billion tons per year. Therefore, a detailed understanding of the formation kinetics and of the position of the ‘nano‐water’ and its storage and dispersion and also its diffusion and percolation options, etc. during the hydrogen‐based reduction process are of high relevance for efficient hydrogen‐based production of sustainable steel (and other metals).

Tackling these questions requires high‐resolution spatial probing of the reaction interfaces and their defect content, high analytical resolution, and real‐space access to the reaction front and in situ‐like experimental conditions.^[^ ^31^, ^32^ ^]^ It must be also considered that the reduction of iron oxides with hydrogen (or other agents) is associated with significant volume changes between the adjacent phases, phase transformations, and high mass loss, effects which all lead to high mechanical stresses, vacancies, nano‐pores, interfacial delamination as well as the formation of multiple types of lattice defects.^[^ ^17^, ^33^, ^34^ ^]^ This means that these redox phenomena are not just of a chemical but also of a structural and mechanical nature.

To study these effects, with particular attention to the location and dispersion of the water, we conducted here a quasi in situ, near atomic‐scale, real‐space study of the hydrogen‐based reduction of iron oxides using a well‐controlled cryogenic and ultrahigh‐vacuum workflow, including also sample and reaction preparation as well as material probing using the latest developments in atom probe tomography (APT).^[^ ^35^, ^36^, ^37^, ^38^, ^39^ ^]^ These features of the experimental workflow are important as water in its nanoscale form is elusive and can get otherwise readily lost, contaminated, or overlooked in such experiments. Our preceding demonstrator experiments have indeed shown that APT analysis of water is possible.^[^ ^40^, ^41^ ^]^ We make the surprising observations that the reaction product is not formed (and removed) as a film on the reaction surface but stored in nanoscale dispersed form at the inner reaction regions and trapped as nanoscale water‐metal inclusions inside the reduced iron.

## 2 Results and Discussion

### 2.1 Key Experiment and Oxygen‐Depleted Reaction Interface

APT specimens are fabricated from single crystalline FeO (wüstite) samples along the crystallographic [100] direction via site‐specific lift‐out in a FEI Helios dual beam Xe‐plasma focused ion beam (FIB) /scanning electron microscope (SEM).^[^ ^42^ ^]^ The APT specimens are then transported into an infrared laser reaction hub module using an ultra‐high vacuum (UHV) transfer suitcase (Ferrovac VSN40S) maintained at cryogenic temperature (liquid nitrogen (LN2) cooled).^[^ ^35^, ^37^, ^43^ ^]^ In this coupled heating and reaction module different APT specimens are exposed to 50 mbar of deuterium gas (D_2_) for 5, 10, 20, 30, and 60 s at 700°C. It is essential to use D instead of H for such experiments, to be able to differentiate and track the intended reaction partners from the artificially acquired hydrogen that can be introduced from the environment and from sample preparation, an effect that can harm such delicate experiments, even under UHV conditions. Before and after heating, samples are kept at the cryogenic temperature of −213.15 °C inside this module. Mass spectrometry is used to verify that only deuterium is present in the reaction chamber. The so‐reduced APT specimens are then transferred through the UHV suitcase to the atom probe instrument at a cryogenic temperature of −140 °C. Details of the instruments and protocols are in Experimental Section.

**Figure 1:**
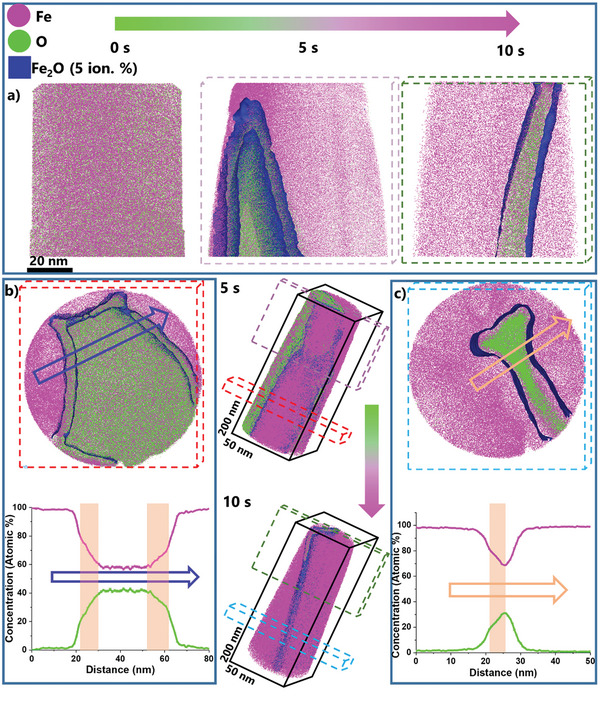
Deuterium‐based reduction of needle‐shaped FeO single crystal specimens: a) APT maps of FeO during successive time steps of reduction at 700°C under 50 mbar deuterium gas atmosphere (D_2_). b) Cross‐section of FeO reduced for 5 s along with a compositional profile showing the Fe—O compositions along the reduction interface. c) Cross‐section of FeO reduced for 10 s along with a compositional profile showing the Fe—O compositions along the reduction interface. The rose box‐shaped shading highlights the oxygen‐depleted interface. Pristine FeO samples show a 52.8 ±0.0197 Fe‐44.3 ±0.0196 O at.% composition. All error values are < 1 at.%. The mass spectrum is shown in Figure S1 (Supporting Information).

APT analysis on pristine FeO (**Figure** **1**a) showed an overall composition of 52.8 ±0.0197% Fe‐ 44.3 ±0.0196 at.% O. We also compare oxygen levels detected at different laser pulsing energies on the same pristine FeO sample, to examine the effect of field conditions on the oxygen content detected. We find, by looking at Figure S2 (Supporting Information), that the oxygen content does vary with laser energy. APT analysis of pure single crystal wüstite shows an under‐stoichiometric oxygen content. This is possibly related to the recombination of oxygen ions at the atom probe tip surface into neutral species, depending on local electrical field strength.^[^ ^44^ ^]^ This effect might lead to the apparent loss of oxygen as the spectrometry only counts charged particles. This was seen in a previous APT study on wüstite reduction.^[^ ^15^ ^]^

The reconstructed APT data show that after only 5–10 s a considerable volume of the specimen has been reduced. Short‐term, interrupted reactions show a gradual increase in reduced Fe, a corresponding decrease in pristine FeO, and an increase in Fe (Figure 1). The reduction interface had been ``frozen in" at different times of the ongoing reaction. After 5 s of reduction at 700°C, regions of pure iron are formed at the surface of the specimen, albeit not covering it fully. The interior of the specimen is composed of an oxide region with an Fe—O (59 at.% Fe‐ 41 at.% O) composition, similar to the (rather wide) composition ranges that have been reported before for wüstite.^[^ ^45^ ^]^ The interfaces between iron and wüstite are characterized by a wide compositional Fe—O gradient, with a gradual drop in oxygen content from ≈ 40 at.% inside the wüstite down to ≈ 1 at.% inside the body‐centered cubic *α*‐Fe over a length of ≈ 12–14 nm, which translates to a length of ≈ 30 wüstite unit cells.

At the transition area between the reduced iron and the non‐reduced oxide, an oxygen‐depleted interface is already developed as clearly revealed in the chemical composition profiles (Figure 1b) (rose box) gathered across the reduction front and perpendicular to the tip direction (Figure 1). The thickness of this interface is found to be directly related to the progression of the reduction. It is also worth noting that we observe a moderate change in the chemical gradient (region in profile marked by rose boxes) when the oxygen content falls below ≈ 18 at.% (left‐hand side of Figure 1b, bottom) or, respectively, 30 at.% (right‐hand side of Figure 1b, bottom). This change in slope might indicate (a) a change in the oxygen diffusion mechanism, (b) a change in the remaining wüstite's structure features, and/or (c) a change in the internal defect and/or pore structure which might alter the oxygen transport. In any case, the data obtained after 5s clearly show that the removal of oxygen from the wüstite is gradual, oxygen diffusion‐mediated, and an altogether rather sluggish process, without producing steep chemical gradients or any similar abrupt composition transitions.

Owing to the varying diameter of the APT specimen's needle shape, the reduced area cross‐section varies along the shank. This means that the reduction happens faster at the top of the specimen, due to its higher surface‐to‐volume ratio. This is evident when comparing the thickness of these reduction interface shells at different distances from the initial specimen's apex, as the thickness of the iron grows from 5 to 10 nm (Figure S3, Supporting Information).

The overall sandwich‐type morphology of the partially reduced sample after 10 s, with only a thin slice of unreduced oxide in the center, indicates that at the fairly low hydrogen partial pressure imposed here, the reduction reaction seems to be at the beginning nucleation‐controlled. This can be also seen from the morphology of the freshly formed iron after 5 s at 700°C (Figure 1), where regions of pure iron are formed at the surface of the specimen, albeit not covering it fully, but only in certain regions. This means that once the iron has nucleated on the surface of the wüstite tip, the inbound growth proceeded until the experiment was interrupted, leaving only a thin slice of unreduced material inside of the tip behind, thus producing the observed sandwich‐type topology. A second aspect that could possibly have contributed to this morphology is that certain crystallographic surface facets of the oxide have higher reduction rates than others, a feature that might also promote the observed anisotropy.

The issue of distinguishing details of the oxygen content in heterogeneous oxide structures by APT arises from the oxygen loss due to the effect of the high field applied during APT probing.^[^ ^44^, ^46^ ^]^ This effect could introduce artefacts that mimic oxygen‐depleted zones in the sample. In our case, we are able to confirm that the oxygen‐depleted interface is indeed due to the reduction process itself. We do that by comparing the reduction at different areas of the reduced sample, and through the analysis of the changes in interface width and composition with progressing reduction. Also, the reduction interface we observe here is parallel to the specimen's main axis, whereas oxygen movement due to applied field is usually driven by the field into the depth of the specimen (an effect which was called field‐induced corrosion in the past^[^ ^47^ ^]^) and perpendicular to the specimen's main axis.^[^ ^48^, ^49^, ^50^ ^]^ Finally, the variation across the reduction interface that we see in our analysis is more significant than that observed when analyzing FeO at different laser energies (Figure S3, Supporting Information). This increases our confidence in the oxygen depletion layer we present in Figure 1.

A similar analysis is obtained for the specimen reduced for 10 s (Figure 1c), where the tip is fully covered by pure Fe, with a remnant oxide region in the center of the tip. The Fe—O ratio in the remaining oxide region is at a maximum of 2.18. More specifically, the oxide composition within the span of 5 nm at the center ranges between 10.6 at.% ± 0.48% and 31 at.% ± 0.31% of oxygen. This finding again reveals that oxygen depletion proceeds gradually. The data also show that the reduction leads to a nanoscale core‐shell morphology, where the outbound diffusing oxygen that leaves the center oxide must either (a) diffuse through the outer iron shell to the specimen's free surface to form deuterated water and/or (b) can form water also inside the specimen, provided that the preceding oxygen mass loss has led to a nano‐pore which can host that water. A clear distinction between the non‐reduced remaining oxide inside the specimen and the reduction interface becomes more difficult at these small dimensions. This applies especially to the thinner region of the specimen, where the metal‐oxide interfaces and the already highly oxygen‐depleted FeO layers beneath begin to overlap, leaving small oxide inclusions behind at advanced stages of reduction.

As an intermediate summary, the APT on‐tip quasi in situ deuterium reduction experiments show that the outbound diffusion of the oxygen creates a wide oxygen gradient inside the wüstite, extending over ≈ 30 crystal cells. The actual removal mechanism of oxygen cannot be resolved at lattice scale, but it is conceivable that the increasing number density of oxygen vacancies, created by the progressing reduction, leads to a moderate acceleration of oxygen transport inside the wüstite.^[^ ^45^ ^]^ A contribution of grain boundaries to the oxygen transport inside wüstite is less plausible because the sample is a single crystal. Deuterium transport is not considered to act as a bottleneck mechanism here, owing to its much higher diffusion coefficient, that is, it may be assumed to be abundant throughout the APT specimen at the temperature and times probed here.

An interesting feature is a change in the depletion slope, which might indicate a change in transport, structure or defects. Among these defects particularly the nano‐pores, that evolve through the collapsing vacancies that the depleted oxygen leaves behind inside the wüstite, deserve particular attention as they might offer locations to host the water, as will be discussed below.

### 2.2 Deuterium Accumulation at the Reduction Interface

The rationale behind using deuterium instead of hydrogen is to differentiate the reactant in our specific reaction from hydrogen typically incorporated in analyses due to the Xe‐plasma FIB preparation^[^ ^51^ ^]^ or atom probe analysis.^[^ ^52^, ^53^ ^]^ In an ideal scenario, we would detect all of our reducing agents as D_2_^+^ at a mass signal of 4 Da to be able to fully distinguish D from H. However, due to the heterogeneity of the sample at these very early stages of the reduction, and the use of laser pulsing in the atom probe tomography experiment, detection of D^+^ comes in peaks of 2 (primarily) and 3 Da (Figure S4, Supporting Information), respectively. Theoretically, H_2_^+^ could also be detected at 2 Da, so in order to confirm that the high signals detected at 2 Da in our experiments can indeed be attributed to the D atom, we carried out a comparison of the ratio of 2 Da counts with respect to those for peaks at 1 and 3 Da for different specimens of Fe run under a range of electrostatic field conditions, indicated by the ratio of Fe^2+^ to total Fe detected in each case, with and without deuterium exposure. As shown in Figure S5 (Supporting Information), the reduced samples in this study do indeed show higher intensity at 2 Da than usual, due to the introduction of the deuterium, confirming that the peak at 2 Da could be treated as a way of measuring the concentration of deuterium reacted during the reduction.

**Figure 2:**
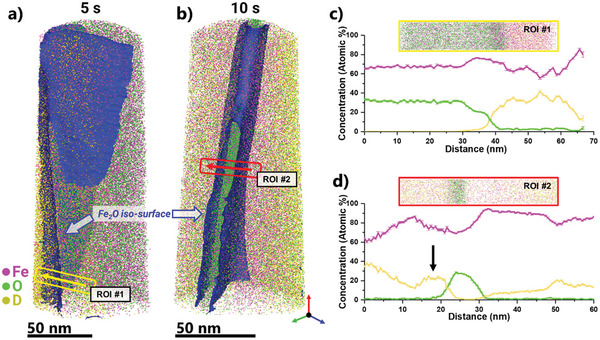
Deuterium at the reduction interface: a) Composition of D^+^ (2 Da) detected at different field conditions for different samples. b) Atom maps of samples in situ reduced for 5 and 10 s at 700°C, respectively. c) Chemical profile across the reduction interface at 5 s. d) Chemical profile across the reduction interface at 10 s.

The atom maps of Fe, O, and D in **Figure** **2**a,b reveal how the D tends to ``attack" the oxide at selected areas, which favors a random depletion assumption as to where the near‐surface reduction of iron oxides actually initiates. As shown in the 5s map (Figure 2a), the reduction front is not completely parallel to the reduction surface. Chemical profiling along one of those areas of D enrichment (Figure 2c), shows that D injection could reach as high as 40 at.%, and that the D atom diffuses inward, after the surface dissociation of the gaseous D_2_.

After 10 s, the compositional profile of D has become more complicated, showing an enrichment right at the reduction interface. Accumulation of D is not seen at the same levels across the sample, confirming again that D reduction proceeds through random sites along the reduction interface at least near free surfaces.

The D sweeps the material very fast due to its high mobility. At least after 10 s at 700°C the D is essentially abundant throughout the material and does not act as a bottleneck to the overall redox reaction. D accumulates at the reaction interfaces/tip surfaces. It may be assumed – but cannot be resolved in the current experiments – that D_2_ first dissociates at the free outer tip surface into 2 D atoms, and enters the material in a single atomic form where it diffuses inbound, first following its chemical potential gradient and then saturating and partitioning among the iron, the wüstite, defects, and the reaction interfaces.

A surprising observation is that the D is not detected within the oxide regions of the samples. Chemical profiles across the 10s reduced sample at multiple heights along the shank of the tip (Figure S6, Supporting Information) confirm that D—when charged at the current pressure levels—does not appear into the oxide within the resolution limits of this experiment. The D signals at the center of the oxide region are practically vanishing in all cases. Interestingly, a plot of the H detected at 1 Da also shows the same trend of partitioning (Figure S7, Supporting Information), highlighting further that the reduction kinetics is controlled by oxygen diffusion to the reduction interface to react with D.^[^ ^23^ ^]^ This behavior could be specific to certain oxides, but not all of them, for example, a previous APT analysis on water‐corroded Zircaloy shows affinity of H‐related species to zirconium oxide.^[^ ^54^ ^]^

Once again, the effect of electrostatic field conditions on H levels detected at 1 Da is investigated using a parameter sweep on FeO APT analysis to ensure minimal impact on our chemical profiles. Figure S8 (Supporting Information) shows that the variation in detected H is within 10% between 60 and 100 pJ. H levels do increase more significantly at 20 pJ, which is probably due to an increase in specimen voltage to maintain the same rate of evaporation (detection rate).^[^ ^55^ ^]^

### 2.3 Where is the Deuterated Water?

**Figure 3:**
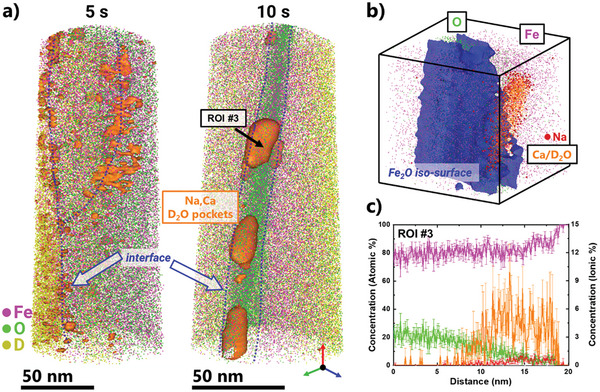
Formation of heavy water droplets: a) Atom maps of FeO reduced for 5 and 10 s, showing the formation and growth of D_2_O clusters. b) A single water nano‐droplet formed after 10 s at the FeO‐Fe interface, along with a chemical profile across showing the high concentration of D_2_O and Na. c) Atomic percentage concentration profiles across a nano‐droplet for Fe, O, and Na, and ionic% concentration profile for Ca‐D_2_O measured at 20 Da. Ca‐D_2_O isoconcentration surfaces are at the value of 4 at.%.

We expect the formation of D_2_O as a by‐product of the reaction of D with FeO. In our work, and due to the fast freezing of samples after reduction, we are indeed able to observe a clear distinct signal at 20 Da indicating the formation of D_2_O as expected (Figure S9, Supporting Information). However, due to the fact that calcium is also detected in the sample analyzed prior to reduction, it may also be that the signal at 20 Da accounts for Ca. Despite the fact that this FeO sample has a much lower impurity content compared to any mineral sample used in real applications, we do still notice several metallic impurities before reduction (including Ca, Al, and Na), as it is very hard to eliminate them completely. Only by using the deconvolution methods in this investigation, we are able to catch the subtle presence of D_2_O. After 5 s of reduction, an atomic scale layer of Ca and D_2_O is seen to form along the reduction interface as revealed in **Figure** **3**a. By looking at the atom map for 10 s, we discover that this layer evolves into clusters (or nano‐droplets) at the reduction interface, as shown in Figure 3a. Since it has been observed that the porosity formed in reduced Fe is due to the escape of water vapor,^[^ ^15^ ^]^ we believe that these nanoscale frozen water droplets form inside such pores that were observed in bulk samples to form during reduction.^[^ ^15^ ^]^

While water is practically electrically non‐conductive, previous studies have shown that water can be analyzed by APT along with incorporated metallic ions.^[^ ^40^ ^]^ In this case, water is rather confined, and it is expected that it would act as a dielectric, based on simulations.^[^ ^56^ ^]^ Another important question is whether these nano‐droplets or, respectively, layers of observed water are liquid or vaporized during the reduction, as the exact confinement and pressure conditions inside such pores are not yet fully understood. Recent studies have shown that confining water to 2–4 nm could decrease its melting point by ≈15%.^[^ ^57^ ^]^ But since the samples are quenched to 80 K (−193.15 °C), and are analyzed at 50 K (−223.15 °C) in the atom probe, we expect that the water is in a frozen solid state during the actual APT analysis.

By focusing on one of the 10 nm clusters formed, we notice further that the clusters of water tend to hold also enriched levels of other metallic impurities, mainly Na (7.14 at. ± % 2.2%). These impurities likely originate from the laboratory‐grown oxide used in this study. This was found also in previous APT analysis on this same material.^[^ ^35^ ^]^ Other impurities are also observed at the water cluster sites, such as Al and N (Figure S10, Supporting Information). Previous work on nanoscale inclusions in geological samples also shows that light metals tend to dissolve in water.^[^ ^58^, ^59^ ^]^ The subsequent analyses of APT specimens reduced for longer times show that tips become fully reduced, while the water‐impurity clusters grow much larger and fully segregated to the surface of the tip at 60s (Figure S11, Supporting Information).

The impurities observed in this study stem in part from the synthesis of the oxide, however, such impurities could in principle also intrude through the so‐called gangue, a term describing elements that occur as natural impurities in oxides used for metallurgical synthesis. This means that our results suggest that at least some of these impurities might play an active role in promoting the formation of confined water. This could motivate further studies on the general relevance of inherited tramp elements on the formation kinetics and dispersion of the encapsulated water.

### 2.4 Why are these Findings About Trapped Nano‐Water Relevant for Sustainable Green Steel Production?

The occurrence of trapped water inside of already reduced metallic material and at the interfaces between the iron and the remaining oxide does not only affect the overall kinetics of the redox reaction but it can even lead to the re‐oxidation of already reduced metallic iron. In a recent investigation, the re‐oxidation of reduced iron due to encapsulated water has been observed by Zhou et al.,^[^ ^29^ ^]^ using a 4D scanning transmission electron microscopy (4D‐STEM) method at a spatial resolution of 1–2 nm and corresponding phase‐field simulations. They studied pores ranging from tens of nanometers to a few micrometers that had been formed by coarsened anion vacancy clusters, that is, in a size region above the atomic‐scale dimensions studied here. The diffraction experiments revealed the formation of single‐crystalline cubic‐Fe_1‐x_O on the inner rims of the pores, although the adjacent volume had been already fully reduced before into iron. The total affected re‐oxidized volume fraction around these pores was estimated to occupy a volume fraction of ≈ 5%. Since the oxidation occurs only in the immediate vicinity of the water‐filled pores, their dispersion influences the total volume affected. In other words, a fine pore dispersion creates a higher volume of re‐oxidized material than a few large pores that occupy the same volume. In an upper‐bound calculation of the corresponding metallization losses (taking a value of ≈ 2 billion tons of steel produced every year as a benchmark), this translates to a value of 100 million tons of iron lost every year.^[^ ^19^ ^]^ In a more realistic estimate, we have to subtract ≈ 1/3rd of that because this is the current global average of steel produced by secondary synthesis, that is, from melting scrap, hence, a more realistic loss value would be 66 million tons per year.

## 3 Conclusion

Using in situ high‐resolution chemical characterization by aid of a well‐controlled cryogenic UVH workflow combining preparation, D‐based reduction reaction, and APT, the reduction of iron oxide by deuterium is now seen at unprecedented chemical and spatial resolution scales.

The reduction starts at the free wüstite surface of the specimens. The gradual depletion of oxygen in the immediate surface regions leads to a gradient in the chemical potential of the oxygen between the highly oxygen‐depleted reaction surface/interface and the interior of the (gradually depleting) wüstite. This gradient in chemical potential leads to outbound diffusion of oxygen, through the newly formed iron layer. The two electrons from the anionic oxygen are taken up by the iron cations, rendering them metallic. These metallic iron atoms then relax and attach to the adjacent metallic iron layer. This elementary step leads to the further inward growth of the iron layer. At the beginning of the redox reaction, recombination between O and D inside of the solid into heavy water will likely not occur, due to the lack of any free volume. This is likely to change as the reaction proceeds further, continuously producing oxygen anion vacancies at the wüstite‐iron interface region. These vacancies can collapse into nano‐pores. These pores could host internally formed heavy water. This means that in principle the water can form not only at the nearest external surfaces but also inside of the pores in the solid.

The analysis of the heavy water confirms that droplets of nano‐confined water are formed at the reaction interfaces, in part even inside of the material, at the hetero–interfaces. This is attributed to the nano‐porosity that hosts the water. It should be also emphasized here that these mechanisms do not coincide but occur in sequence, that is, first the D sweeps the material; second, the oxygen diffuses outbound, leaving behind vacancies and nano‐pores; and third, water can form inside of these pores or at the free tip surfaces. Furthermore, the absence of D from the oxide phase suggests further how useful oxide layers might be in acting as a shield against forms of corrosion that are initiated by hydrogen diffusion.

## 4 Experimental Section

### Single‐Crystalline FeO Samples

To start with a model specimen, a single crystal wüstite sample oriented toward the [100] direction, (an orientation accuracy of <0.1° to 0.05°) across its thickness was used. The sample was lab grown through the Czochralski (CZ) method, provided by Mateck GmbH. In the CZ method, the oxide was formed by inserting a small seed crystal into an oxide melt in a crucible, pulling the seed upward to obtain a single crystal. Thus, eliminating factors such as impurities and porosity that might be found in ore pellets. The reduction protocol and preliminary obtained results are discussed further.

### FIB/PFIB Preparation

Specimens were mounted on a laser‐ablated cold‐rolled 304 stainless steel (304SS) TEM half‐grid (sourced from JPT and CAMECA) following recently developed protocols.^[^ ^35^ ^]^

### Atom Probe Tomography

Atom probe tomography (APT) analysis was performed on pristine FeO to measure the detected compositions of Fe in ratio to oxygen and to measure impurity compositions. A parameter sweep was employed to compare the Fe/O ratios before and after reduction.

Cameca LEAP 5000 XR was used for all reduced APT tips. APT experiments for reduced FeO specimens were conducted in laser‐pulsed mode, with laser energy of 50 and 70 pJ, pulse frequency of 200 kHz, set point temperature of 45 K (−228.15 °C), and 1% detection rate. Data reconstruction and analysis were carried out using AP Suite 6.0.

APT specimens in the direction perpendicular to the thickness of the FeO substrate [100] direction were prepared via standard site‐specific lift‐out procedure^[^ ^36^ ^]^ using a FEI Helios dual beam Xe‐plasma FIB/SEM. UHV carry transfer suitcases (Ferrovac VSN40S) were employed^[^ ^37^ ^]^ for the current study. Cryogenic temperatures were maintained inside the suitcases by liquid nitrogen. The suitcases were designed to hold a modified Cameca APT puck and have a 50‐cm long wobble stick which ends with a PEEK‐insulated puck manipulator. A pressure of 10^−10^ mbar could be achieved inside suitcases. The suitcase could be mounted onto the experimental platforms through specially designed load locks (Ferrovac VSCT40 fast pump‐down docks), pumped via a 80 L s^−1^ turbopump (Pfeiffer HiPace 80).

Deconvolution of peaks at 20‐21‐22 Da, was done using the APsuite software.^[^ ^38^ ^]^ This confirmed that the isotope abundances do not completely match the measured intensity at 20 Da. Our analysis shows that ≈1000 ions could not be attributed to Ca and were instead probably due to D_2_O. In agreement with ex situ APT studies done before on reduced iron oxide ores^[^ ^15^, ^39^ ^]^ metallic impurities could partition or accumulate at the reduction interface.

### Reaction Hub Measurements

In line with previously reported protocols, an IR laser in the reaction hub module was used to heat samples at the following times: 5, 10, 20, 30, 60, and 120 s. All of these heating treatments were performed in an environment of 50 mbar of deuterium. Before and after heating, samples were kept at a cryogenic temperatures of 60 K (−213.15 °C), using the cryo‐chiller. Mass‐spectrometry (Figure S12, Supporting Information) was used to verify that only deuterium was present in the reaction chamber, as the presence of hydrogen could complicate analysis, and presence of water vapor could cause condensation on APT tips which were proven to deter reaction. The sample was then transferred through the UHV suitcase to the atom probe. Figure S13 (Supporting Information) shows the main components in the reacthub module.

## Conflict of Interest

The authors declare no conflict of interest.

## Supporting information

Supporting Information

## Data Availability

The data that support the findings of this study are available from the corresponding author upon reasonable request.
