# Supplementary material for: The Fate of Water in Hydrogen‐Based Iron Oxide Reduction
Source: Adv Sci (Weinh). 2023 Jun 8;10(24):2300626. doi: 10.1002/advs.202300626 (PMC10460863; doi:10.1002/advs.202300626)
Supplement: Supplementary file 1 — Supporting Information [file ADVS-10-2300626-s001.pdf]

ADVANCED SCIENCE
Open Access

Supporting Information

for *Adv. Sci.*, DOI 10.1002/advs.202300626

The Fate of Water in Hydrogen-Based Iron Oxide Reduction

Ayman A. El-Zoka, Leigh T. Stephenson, Se-Ho Kim, Baptiste Gault and Dierk Raabe**

The fate of water in hydrogen-based iron oxide reduction

A.A. El-Zoka *, L.T. Stephenson, S.–H. Kim, B. Gault, D. Raabe*

[a] Max-Planck-Institut für Eisenforschung, Max-Planck-Strasse 1, 40237, Düsseldorf, Germany

[b] Department of Materials, Royal School of Mines, Imperial College, London SW7 2AZ, United Kingdom

* Corresponding Authors

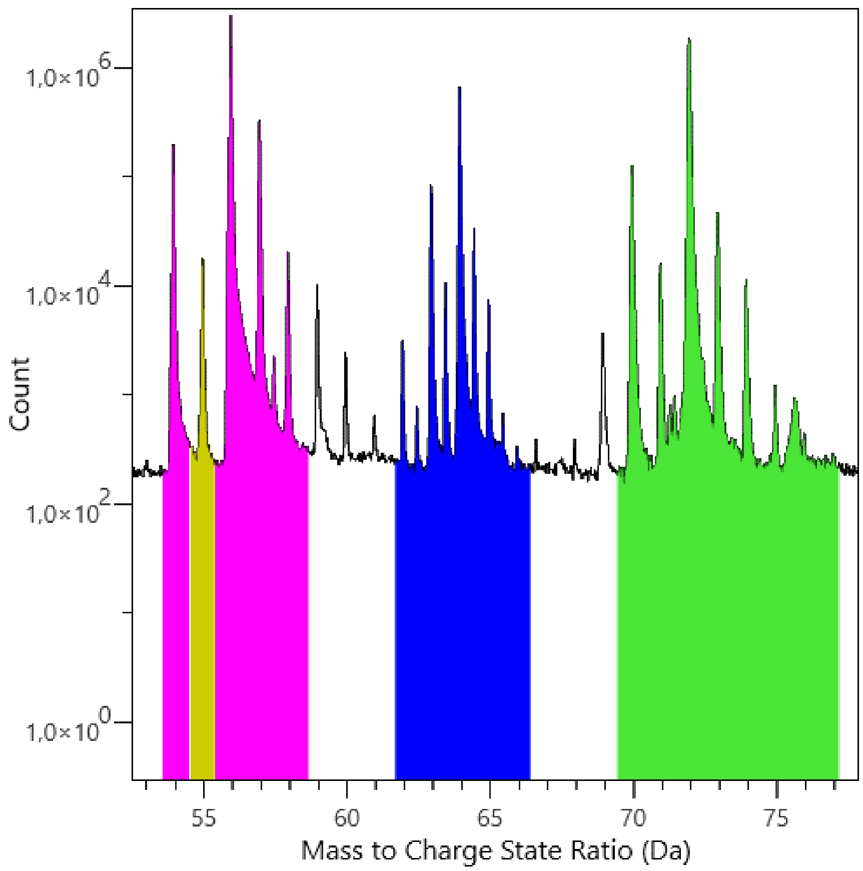

Figure S1. A partial mass-spectrum for the 10s data, showing, the detected Fe (purple), Fe_2O (blue), and FeO (green).

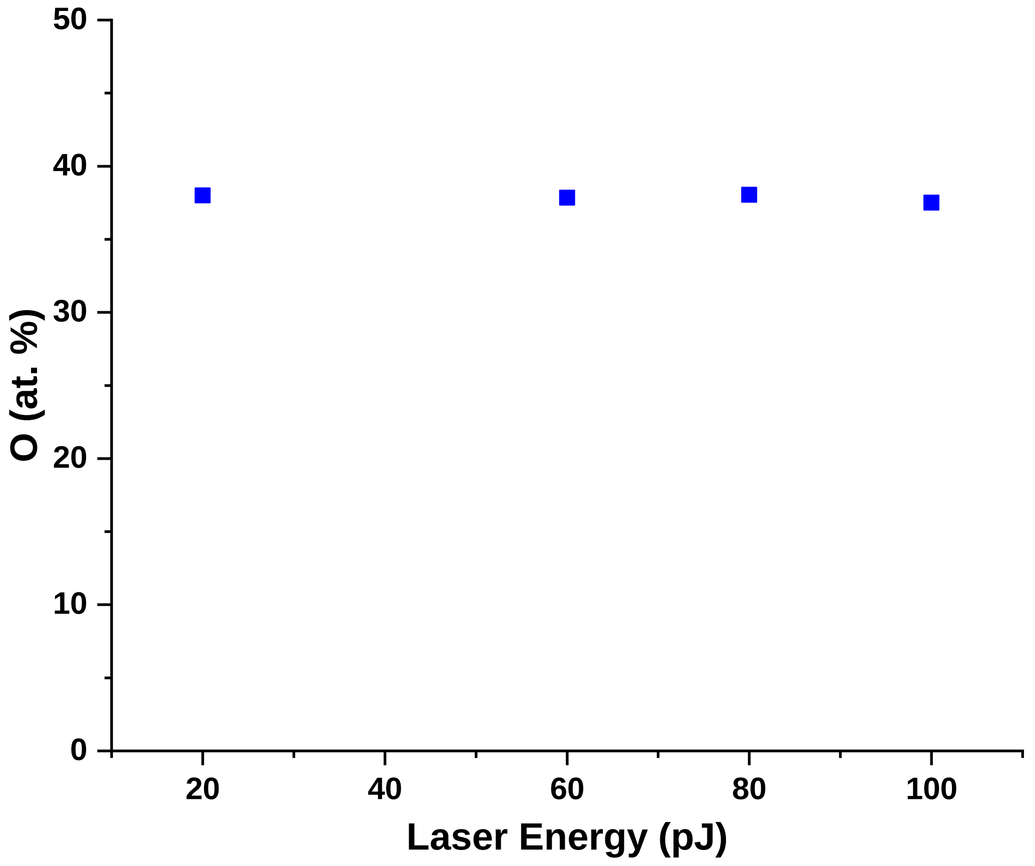

Fig S2. Oxygen composition, detected at different pulsing energies.

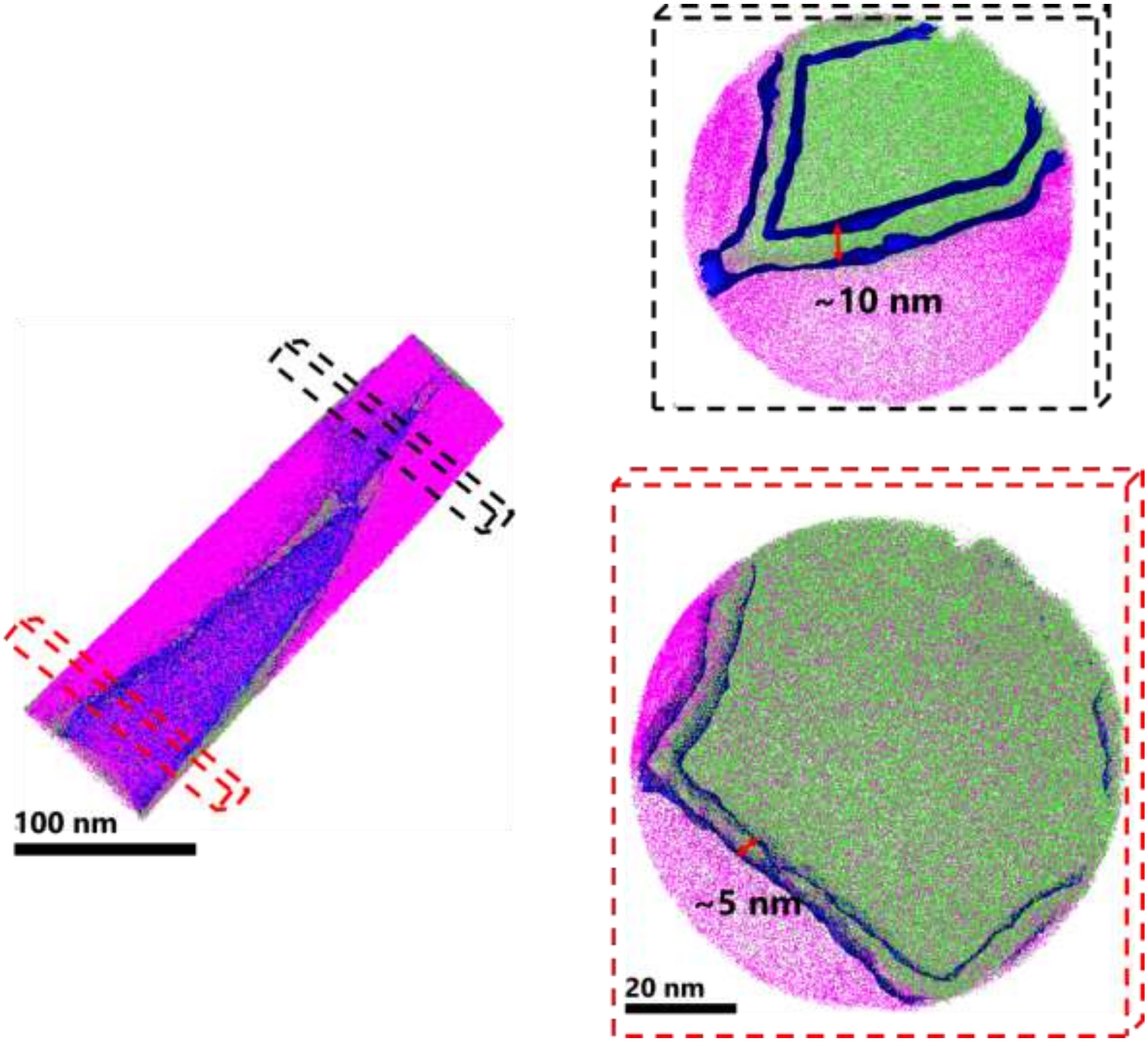

Figure S3. Effect of tip radius on the local progression of reduction. Comparison of reduction interfaces at two different lengths along the tip.

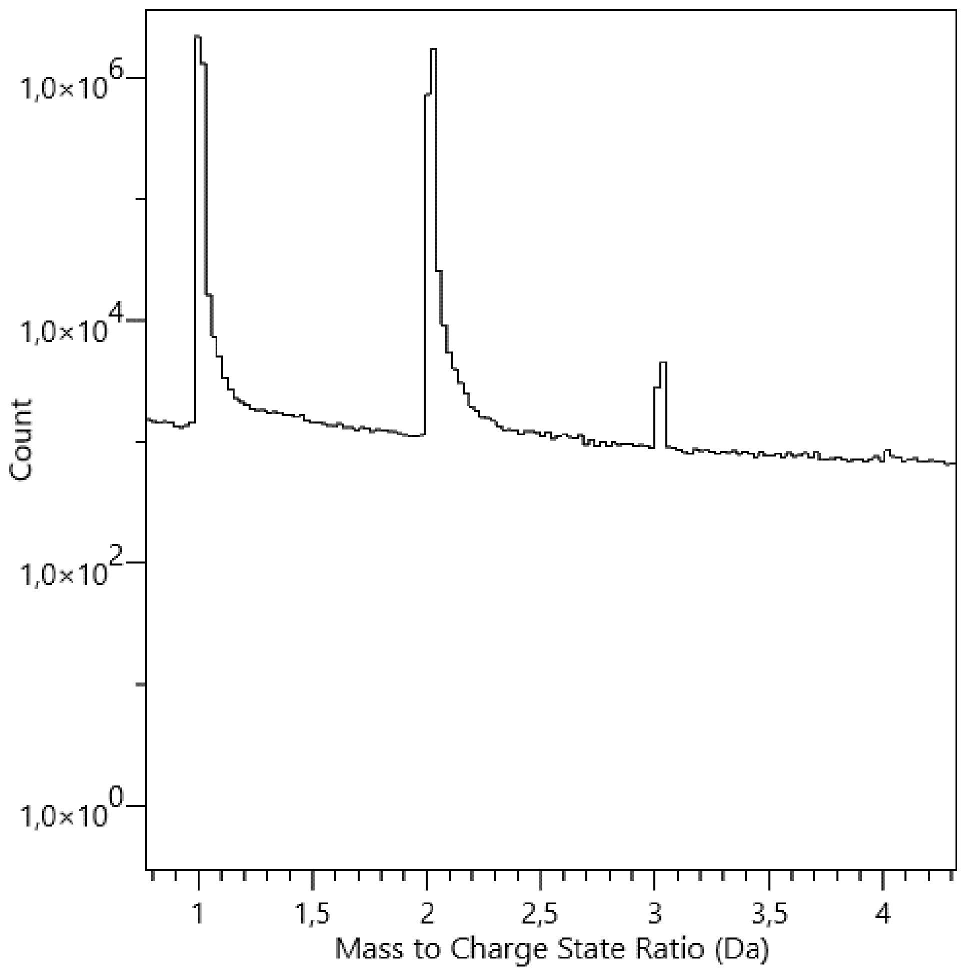

Figure S4. A partial mass-spectrum for the 10s data, showing, peaks at 1 Da (H), 2 Da (H_2 or D), and 3 Da (DH).

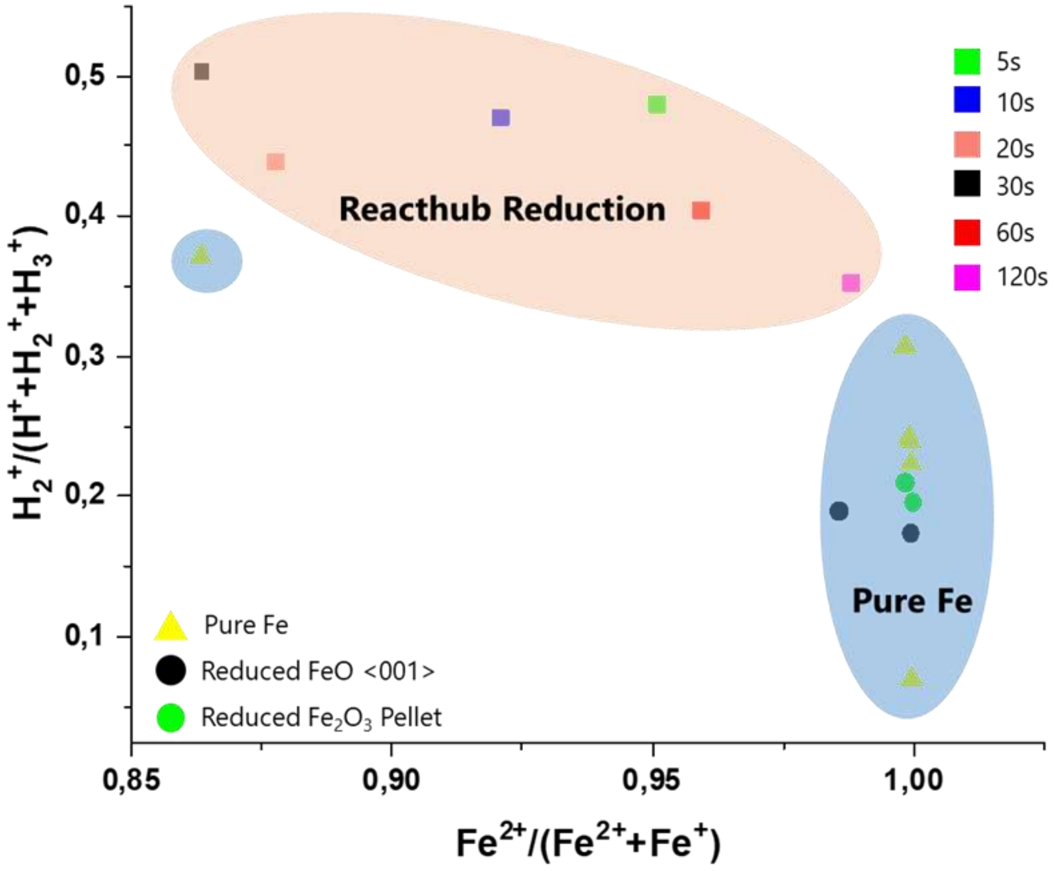

Figure S5. A comparison of peak count ratio for H_2 between samples reduced and frozen, and others including pure untreated Fe, FeO reduced and transferred at room temperature, and natural Fe_2O_3 reduced and transferred at room temperatures. All frozen deuterated samples show a clear consistent trend of having higher than expected counts for the peak at 2 Da.

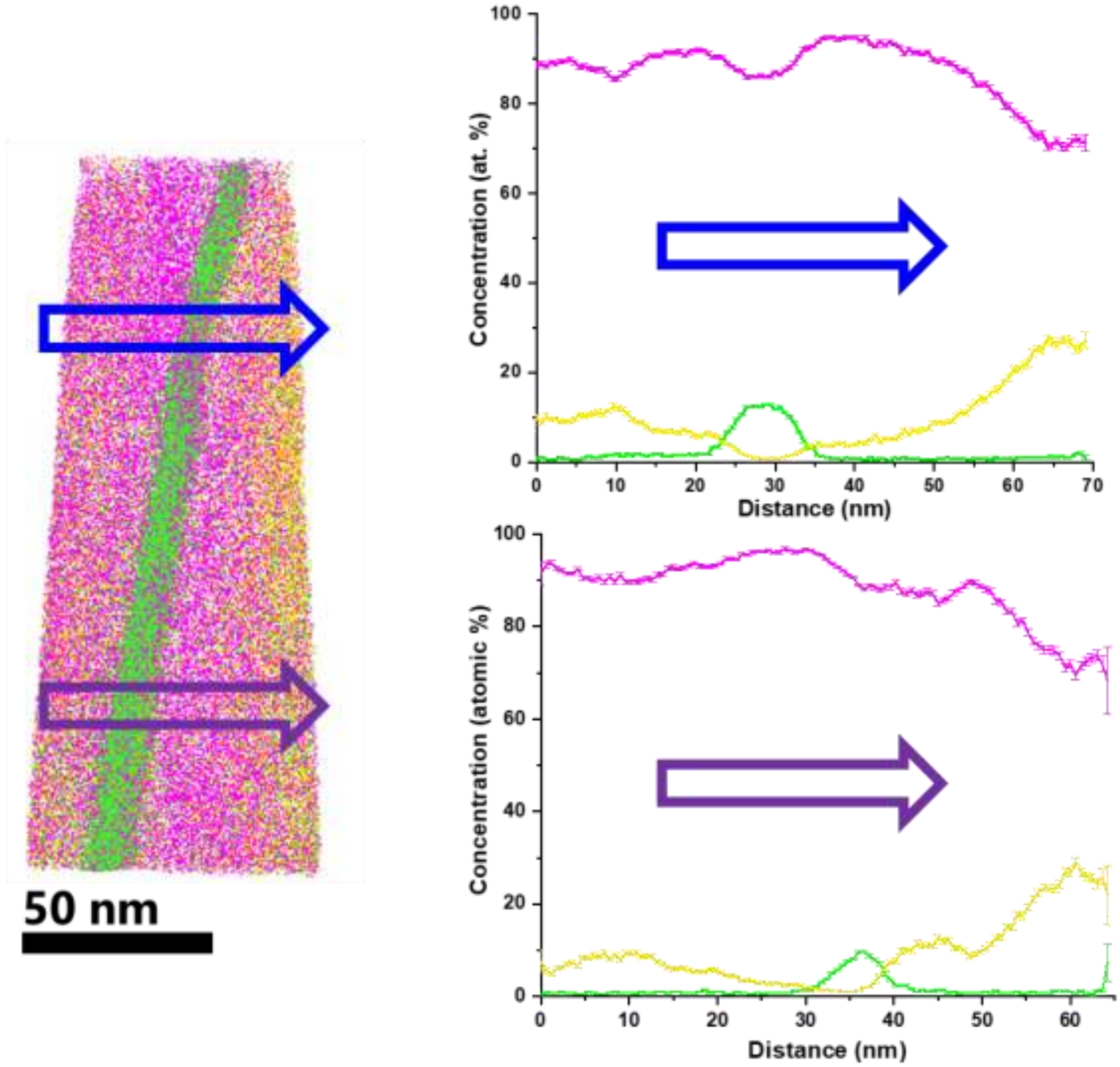

Figure S6. Chemical profile across the reduction interface at 10 s along different locations in the APT sample.

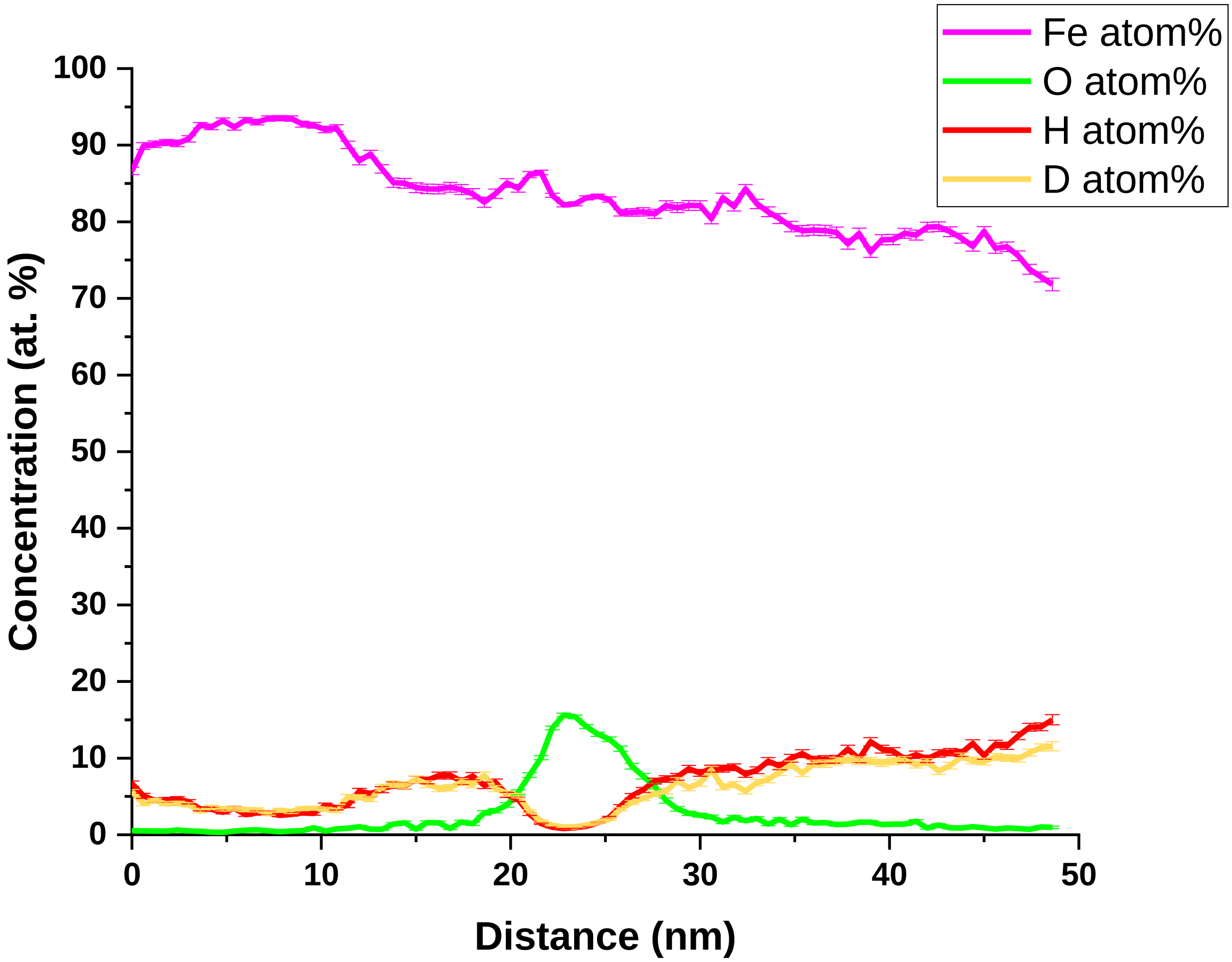

Fig S7. Oxygen composition, detected at different pulsing energies.

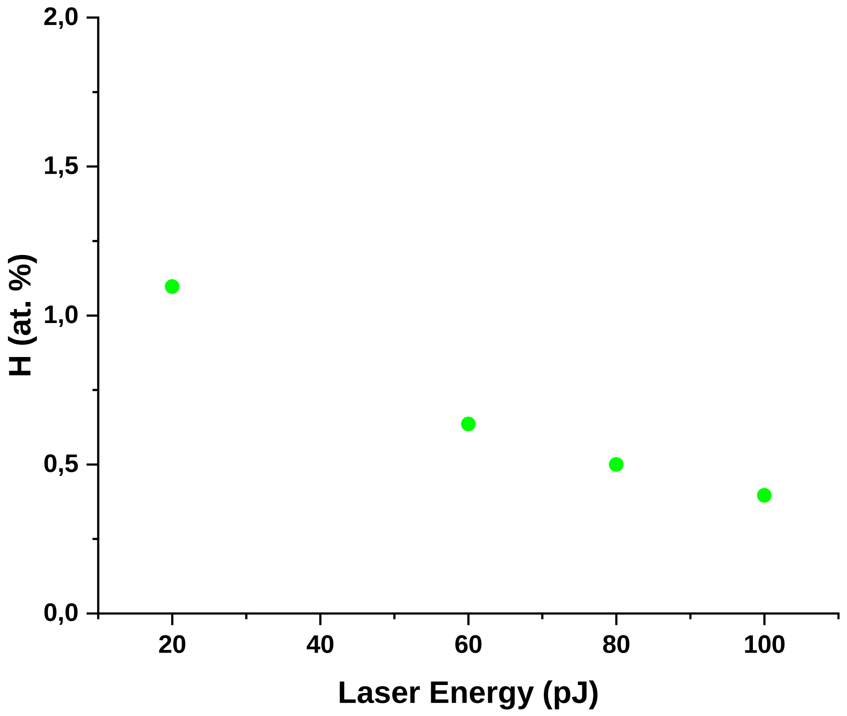

Fig S8. H at. % composition, detected at different pulsing energies.

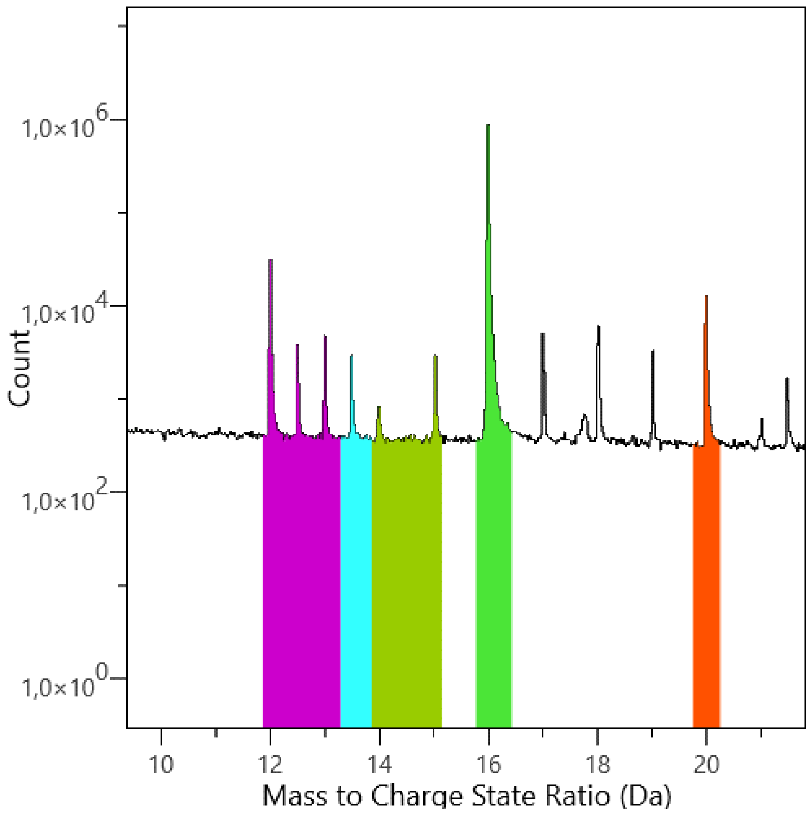

Figure S9. A partial mass-spectrum for the 10s data, showing signals detected for Mg (purple), Al (light blue), N (light green), O (green), and D_2O (red).

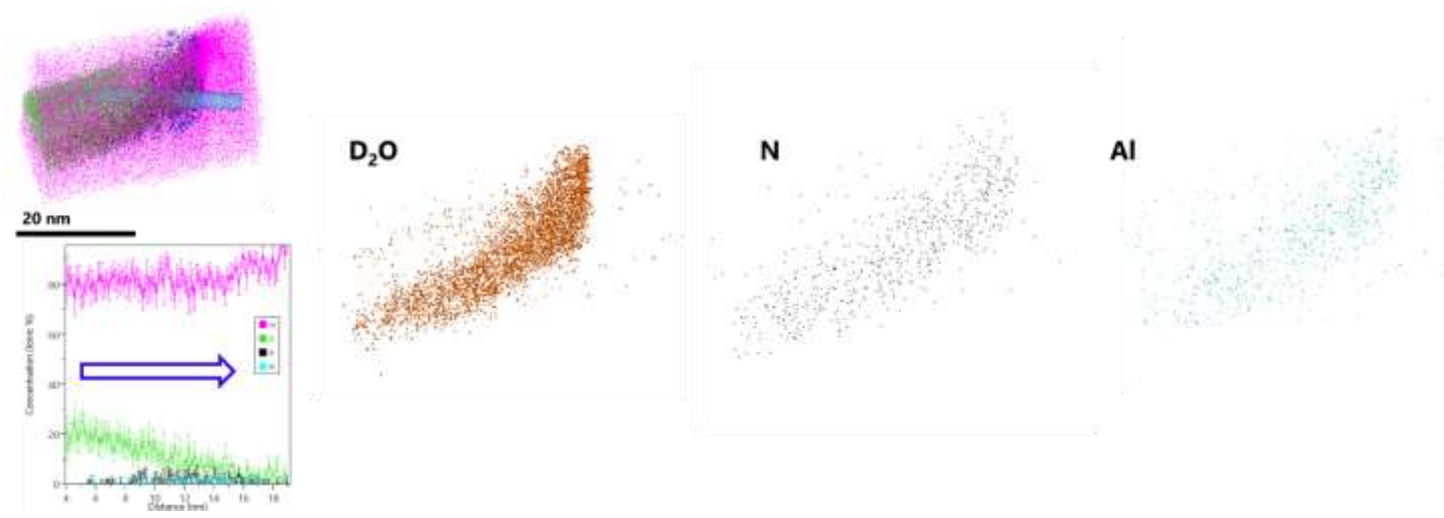

Figure S10. Local enrichment of impurities such as, N and Al at Ca-D_2O clusters formed after 10 seconds of reduction.

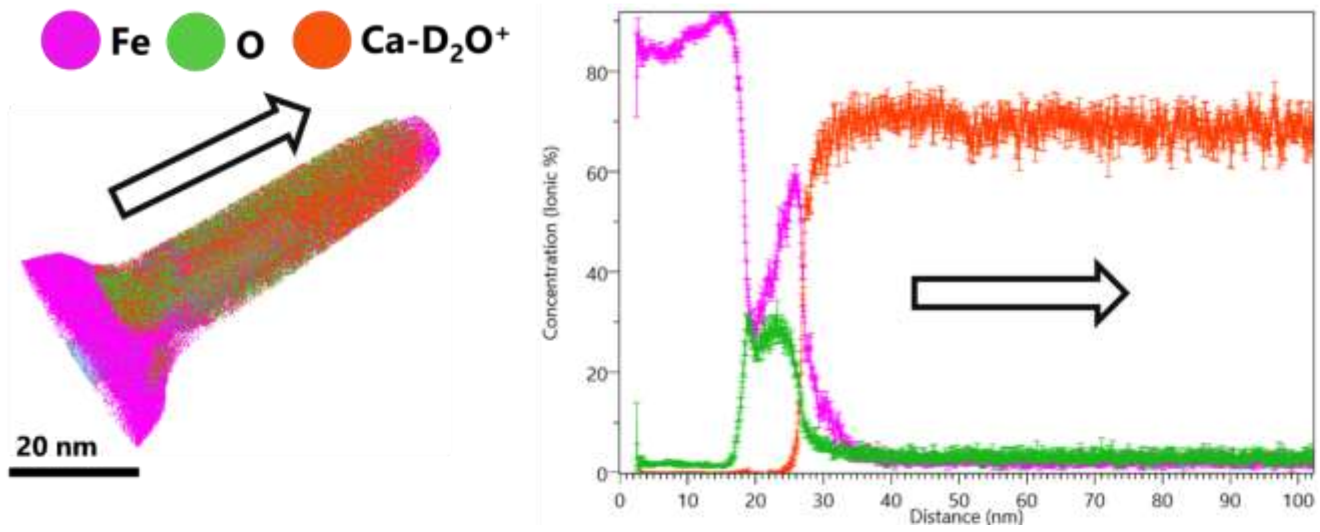

Figure S11. Growth of water-metal droplets after 60s of reduction.

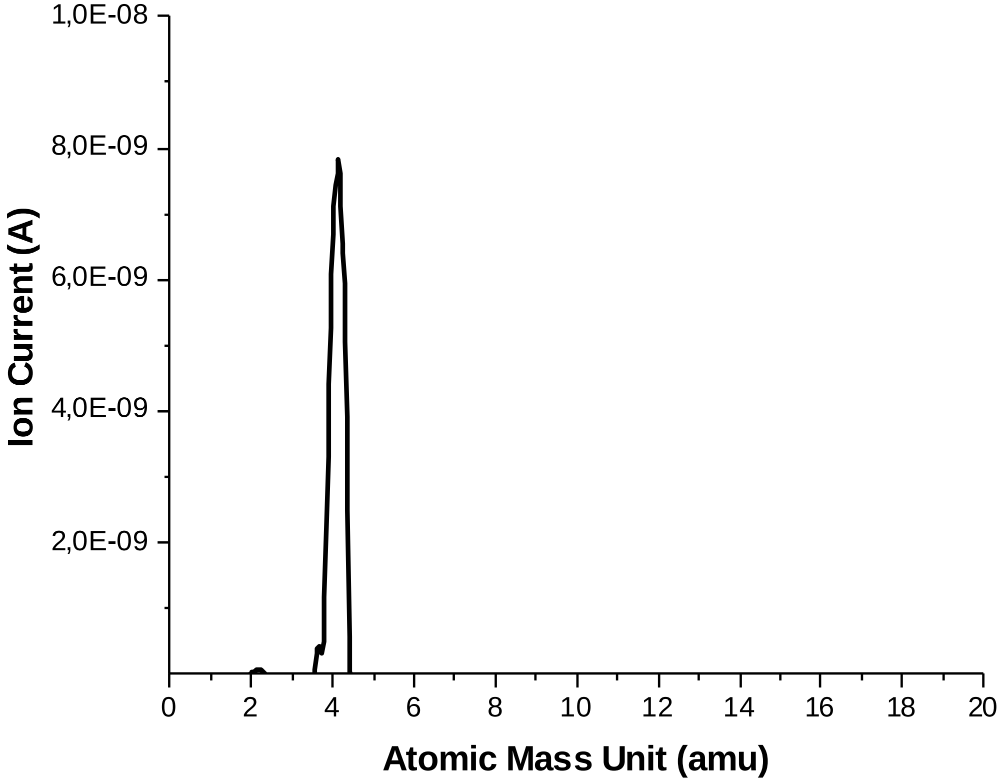

Figure S12. Gas-phase mass spectrometry of the environment inside the reacthub reaction chamber, confirming the presence of deuterium only. Ion currents measured indicate intensity. Main peak detected is at 4 amu (D_2) and a minor peak (max. intensity at E-11 A) was detected at 2 amu (D).

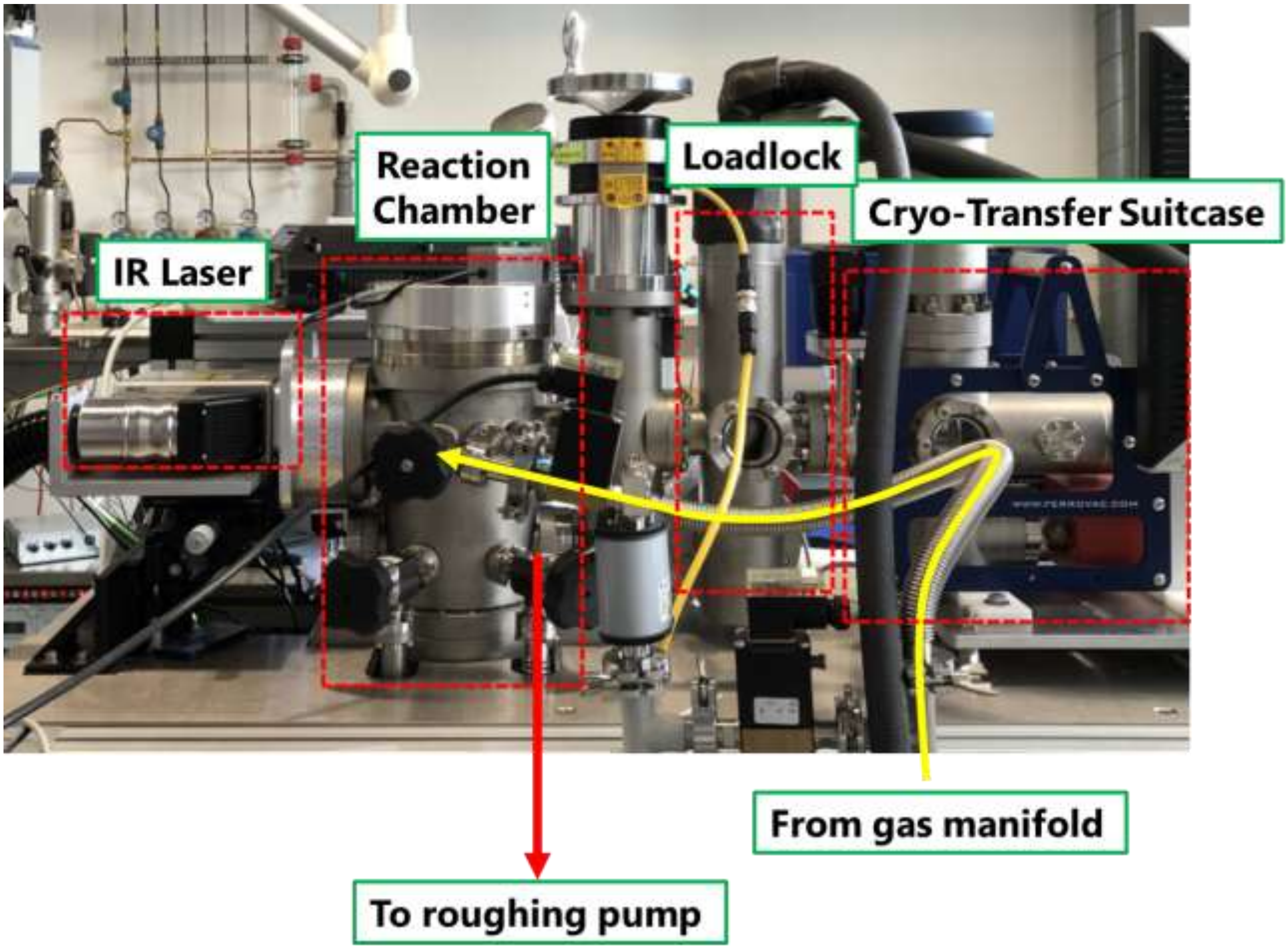

Figure S13. The main components to the reacthub module, more technical details are discussed thoroughly in a recent publication [20].
